# Artificial intelligence driven clustering of blood pressure profiles reveals frailty in orthostatic hypertension

**DOI:** 10.1113/EP091876

**Published:** 2024-11-11

**Authors:** Claire M. Owen, Jaume Bacardit, Maw P. Tan, Nor I. Saedon, Choon‐Hian Goh, Julia L. Newton, James Frith

**Affiliations:** ^1^ Population Health Sciences Institute, Faculty of Medical Sciences Newcastle University Newcastle upon Tyne UK; ^2^ Interdisciplinary Computing and Complex BioSystems (ICOS) research group, School of Computing Newcastle University Newcastle upon Tyne UK; ^3^ Ageing and Age‐Associated Disorders Research Group, Department of Medicine, Faculty of Medicine Universiti Malaya Kuala Lumpur Malaysia; ^4^ Centre for Innovations in Medicine Engineering Universiti Malaya Kuala Lumpur Malaysia; ^5^ Department of Medical Sciences, School of Medical and Life Sciences Sunway University Bandar Sunway Malaysia; ^6^ Division of Geriatric Medicine, Department of Medicine, Faculty of Medicine Universiti Malaya Kuala Lumpur Malaysia; ^7^ Department of Mechatronics and Biomedical Engineering, Lee Kong Chian Faculty of Engineering and Science Universiti Tunku Abdul Rahman Kuala Lumpur Malaysia; ^8^ Health Innovation North East and North Cumbria, Gallowgate Newcastle upon Tyne UK; ^9^ Falls and Syncope Service Newcastle upon Tyne Hospitals NHS Trust Newcastle upon Tyne UK

**Keywords:** ageing, blood pressure, cardiovascular physiology, cognition, frailty, haemodynamics, orthostatic hypertension, orthostatic hypotension

## Abstract

Gravity, an invisible but constant force , challenges the regulation of blood pressure when transitioning between postures. As physiological reserve diminishes with age, individuals grow more susceptible to such stressors over time, risking inadequate haemodynamic control observed in orthostatic hypotension. This prevalent condition is characterized by drops in blood pressure upon standing; however, the contrary phenomenon of blood pressure rises has recently piqued interest. Expanding on the currently undefined orthostatic hypertension, our study uses continuous non‐invasive cardiovascular data to explore the full spectrum of blood pressure profiles and their associated frailty outcomes in community‐dwelling older adults. Given the richness of non‐invasive beat‐to‐beat data, artificial intelligence (AI) offers a solution to detect the subtle patterns within it. Applying machine learning to an existing dataset of community‐based adults undergoing postural assessment, we identified three distinct clusters (iOHYPO, OHYPO and OHYPER) akin to initial and classic orthostatic hypotension and orthostatic hypertension, respectively. Notably, individuals in our OHYPER cluster exhibited indicators of frailty and sarcopenia, including slower gait speed and impaired balance. In contrast, the iOHYPO cluster, despite transient drops in blood pressure, reported fewer fallers and superior cognitive performance. Surprisingly, those with sustained blood pressure deficits outperformed those with sustained rises, showing greater independence and higher Fried frailty scores. Working towards more refined definitions, our research indicates that AI approaches can yield meaningful blood pressure morphologies from beat‐to‐beat data. Furthermore, our findings support orthostatic hypertension as a distinct clinical entity, with frailty implications suggesting that it is worthy of further investigation.

## Abstract (highlights)

- **What is the central question of this study?**
  Can blood pressure morphologies akin to those found in dysregulated postural blood pressure conditions be identified from continuous non‐invasive cardiovascular data using artificial intelligence?
- **What are the main findings and their importance?**
  The findings revealed differences in frailty phenotypes between morphologies indicative of orthostatic hypotension and orthostatic hypertension, with increased frailty being associated with orthostatic hypertension. These results add to the emerging evidence for the clinical significance of the currently undefined condition of orthostatic hypertension.

## 1 INTRODUCTION

Clinicians typically rely on intermittent sphygmomanometer readings to identify dysregulated postural blood pressure, observing sporadic changes when an individual moves from a seated or supine position to standing. However, the current consensus for diagnosing initial orthostatic hypotension necessitates the use of continuous measurement because it requires blood pressure to be captured within 15 s of standing upright. Beat‐to‐beat technology is also subtly endorsed for classic orthostatic hypotension, as indicated by the inclusion of the term ‘sustained’ in its diagnostic criteria. The guidance does not clarify what constitutes a ‘sustained’ trend in blood pressure, and although intermittent sphygmomanometry fails to detect brief fluctuations or ongoing trends, continuous non‐invasive measurement can significantly raise the rates of orthostatic hypotension, as demonstrated in a systematic review and meta‐analysis, with fourfold prevalence found when using this technology (Saedon, Pin Tan, & Frith, 2020).

The association between orthostatic hypotension and increased risk of frailty and occurrence of falls is well established (Finucane, O'Connell, Donoghue, et al., 2017; Mol et al., 2019; Romero‐Ortuno, Cogan, Foran, et al., 2011, Romero‐Ortuno, Cogan, O'Shea, et al. 2011), but attention is now turning to orthostatic hypertension owing to emerging evidence of adverse outcomes. Given the association of orthostatic hypotension with frailty, it is plausible that the reverse might be true for orthostatic hypertension. Owing to the present lack of a consensus definition for orthostatic hypertension, diagnosis and research mirror the thresholds for classic orthostatic hypotension, but in the opposite direction, evaluating increases in blood pressure instead. The consensus criteria (Freeman et al., 2011) for the diagnosis of orthostatic hypotension are as follows: initial orthostatic hypotension involving transient drops of ≥40 mmHg systolic blood pressure (SBP) and/or ≥20 mmHg diastolic blood pressure (DBP) within the first 15 s of standing upright and classic orthostatic hypotension occurring with sustained decreases of ≥20 mmHg SBP and/or ≥10 mmHg DBP within 3 min of standing.

The haemodynamic response to postural change can provide insight into overall physiological health and is especially relevant to the function of the cardiovascular and autonomic nervous systems. Continuous non‐invasive cardiovascular monitoring offers a deeper analysis throughout the positional change, capturing beat‐to‐beat blood pressure details that intermittent methods might miss. Grouping study participants based on crude thresholds at fixed time points is likely to omit substantial detail, falling short of encompassing the entire spectrum of postural blood pressure profiles. Artificial intelligence (AI) steps in at this juncture, being adept at processing large‐volume data and exposing multidimensional patterns within it. It represents a currently untapped resource in investigating orthostatic hypertension, potentially revealing aspects that might have been overlooked previously owing to the focus solely on blood pressure deficits.

This study uses AI to distinguish clusters of postural blood pressure morphology from continuous non‐invasive cardiovascular data, subsequently evaluating the differences in clinical measures of frailty markers among them.

## 2 MATERIALS AND METHODS

Data were sourced from the Malaysian Elders Longitudinal Research (MELoR) study. Procedures and protocols were approved by the University of Malaya Medical Centre Medical Ethics Committee (MEC: 943.6) in compliance with the standards set by the latest revisions of the 1975 *Declaration of Helsinki*. Written informed consent was obtained from all participants prior to their participation.

### 2.1 Study population and design

The MELoR study is a cohort study situated in Kuala Lumpur and its neighbouring Klang Valley suburbs. The MELoR study includes urban community‐dwelling residents aged ≥55 years. The age criterion for inclusion was chosen to coincide with the retirement age in Malaysia at the time the study was conceived. Participants representing Malay, Chinese and Indian ethnic groups were selected through random stratified sampling from the electoral rolls of the parliamentary constituencies of Petaling Jaya North, Petaling Jaya South and Lembah Pantai. MELoR adopted a multidisciplinary methodology, using computer‐assisted interviews at participants’ homes and clinically validated assessments conducted at hospitals. These evaluations included anthropometric measurements, laboratory tests and assessments of both physical and cognitive performance. Exclusion criteria were bed‐bound individuals unable to attend hospital assessments and those with severe impairment of communication. From the 1411 complete health assessments in the first wave, a dataset of 1135 beat‐to‐beat cardiovascular recordings was collated.

### 2.2 Haemodynamic assessment

The beat‐to‐beat cardiovascular data were recorded using a Task Force Monitor (Task Force, CNSystem, Austria; Fortin et al., 2006), incorporating digital photoplethysmography and vascular unloading technology. The default position of the finger v‐cuff was the proximal phalanx of the middle finger of either hand, relocated to the index finger if required, for improved signal quality. The hand and forearm were secured at heart level using a customized arm sling, and the contralateral arm was fitted with a size‐matched oscillometric blood pressure cuff. This provided the intermittent brachial artery pressures against which the local finger pressures were calibrated and amplified.

The beat‐to‐beat cardiovascular recordings underwent screening through custom‐designed algorithms within a signal quality framework developed specifically for this task. This process involved inspecting signals for poor quality and identifying and excluding those with anomalies such as noise, artefact or calibration errors, which were reflected in marked deviations from expected trends.

Participants underwent the conventional ‘active stand test’, which involved continuous haemodynamic monitoring throughout the transition from supine to standing upright. The objective was to achieve this positional shift within a time frame of 3 s. In the 10  min supine phase, a baseline was established as a comparative reference point to measure relative postural blood pressure shifts. The baseline was determined by averaging the readings 60–30 s before assuming the upright position. This period was selected to create a stable baseline that minimized motion‐related noise and avoided anticipatory blood pressure rises that could occur closer to the initiation of standing (Finucane et al., 2019).

The standing phase was recorded for 3 min; however, for this study, the observation window was narrowed to the first 2 min as per research in the field (Romero‐Ortuno, Cogan, Foran, et al., 2011). Additionally, this approach maintained data integrity, considering instances when active stand tests were concluded prematurely and in end‐of‐test phases susceptible to noise introduced by participant movement. The decision to focus the clustering model solely on SBP changes stemmed from the prevailing trend in research of dysregulated postural blood pressure, which prioritizes SBP over DBP. This approach is supported by the finding by Fedorowski et al. (2017) that 95% of classic orthostatic hypotension cases are identifiable through SBP changes alone. In the proposal for orthostatic hypertension consensus, panel experts suggest that rises of DBP upon standing might be spurious, advocating instead for the use of SBP (Jordan et al., 2023).

### 2.3 Clustering model

**FIGURE 1:**
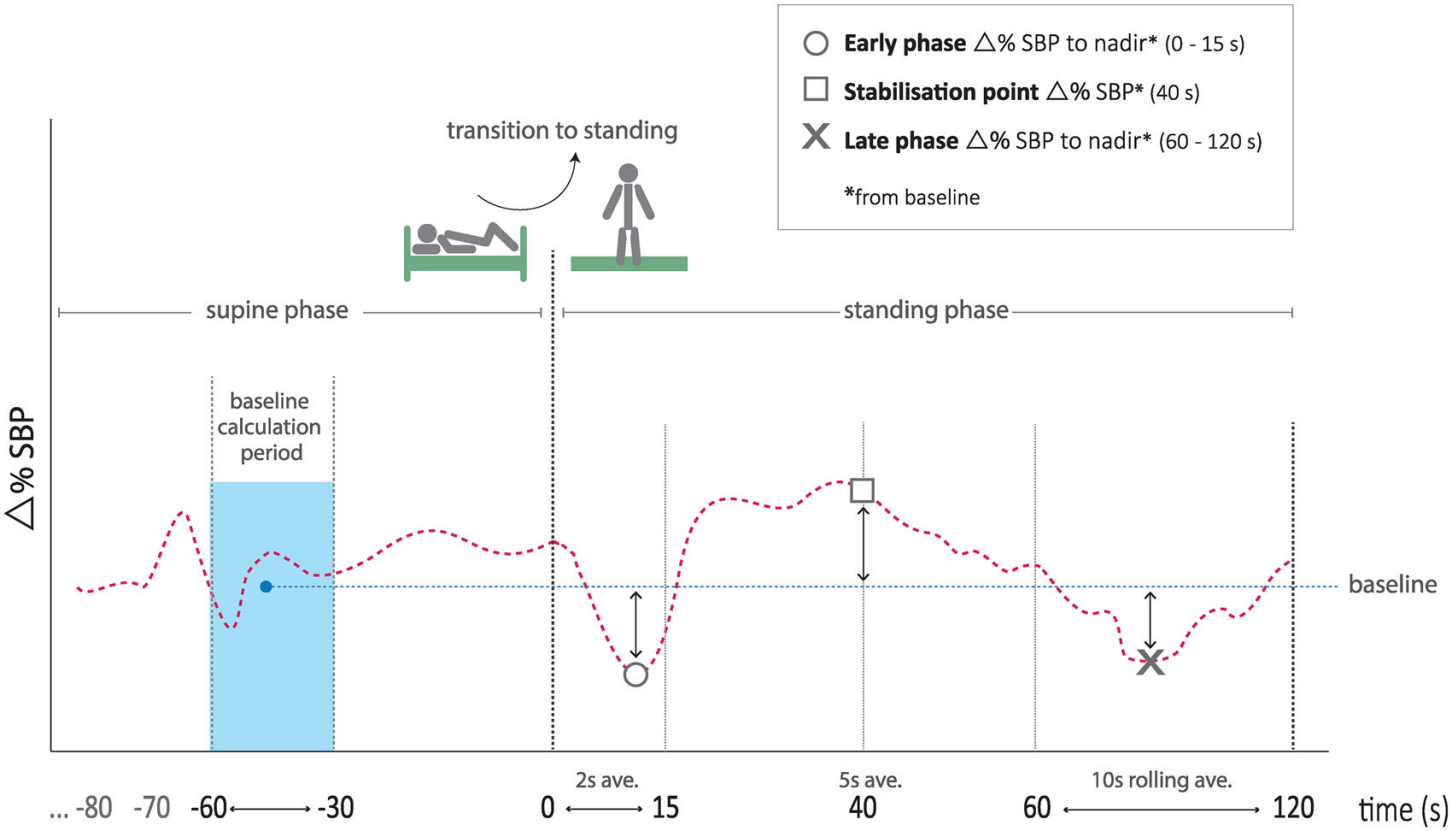
Illustrative example of beat‐to‐beat monitoring during the active stand test with selected postural blood pressure features: early phase, stabilization point and late phase for the clustering model. Features are derived from the magnitude of shift from baseline (Δ% SBP). The period for baseline calculation was taken during the supine phase, shaded in blue. Active stand phase duration was from 0 to 120 s. Abbreviation: SBP, systolic blood pressure.

Continuous non‐invasive cardiovascular data were sampled with each heartbeat. To facilitate extraction of features for clustering, interpolated values were computed to create a uniform 1‐s interval sample rate. Data from three time points were extracted from the beat‐to‐beat SBP recordings of each participant. Corresponding to their associated periods during the active stand test, these features were termed ‘early phase’, ‘stabilization point’ and ‘late phase’, as illustrated in Figure 1. The criteria and time points for the features in our study were determined based on their individual or combined morphologies, reflecting various characteristics of orthostatic hypotension: the ‘early phase’ representing initial orthostatic hypotension, the ‘stabilization point’ as a marker of impaired early stabilization and the ‘late phase’ aligning with the consensus criteria for sustained deficits.

To quantify the extent of SBP shift from baseline at these three feature points, the calculation was based on the percentage difference from the lowest SBP value (nadir point) within the first 15 s of standing, the percentage difference SBP reading at 40 s and the lowest SBP range (nadir period) calculated from 10‐s rolling averages between 60 and 120 s. Two‐second and 5‐s averaging were applied at the ‘early phase’ and ‘stabilization point’, respectively.

The *k*‐means++ algorithm was implemented through the scikit‐learn library (RRID:SCR_019053). A machine learning toolkit in the Python Programming Language (v.3.8.10, RRID:SCR_008394) was used for the clustering analysis. Feature vectors derived from early phase, stabilization point and late phase criteria were extracted from each participant's postural blood pressure recording. The model was executed with the specified number of clusters, *k* = 3. The optimum value of *k* was selected by assessing cluster separation with the silhouette score obtained for differing *k*. Additionally, the number of iterations required for convergence, and the Jaccard similarity coefficient from multiple bootstrapped samples were consulted to assess the stability and consistency of the clusters.

### 2.4 Normal orthostatic response group

We established a normal orthostatic response (NOR) reference group to assess frailty across the clusters, using the same three feature points as those in our clustering model. In this reference group, a ‘normal’ orthostatic response meant an SBP change within 20% of the baseline in the early phase and within 10% at both the stabilization point and late phase. A greater margin in the early phase was set to accommodate the known increase in blood pressure variability during the initial 15 s of standing. During this phase of the analysis, participants who met these criteria at all three time points were classified into the NOR reference group from their original clusters for the frailty comparison.

For formation of the NOR reference group, 254 participants were identified as eligible for reassignment from their original clusters to the NOR group. This reassignment resulted in changes to the cluster sizes: the iOHYPO cluster was reduced by 51.5% from 431 to 209 participants, there was no change in the OHYPO cluster, and the OHYPER cluster was reduced by 13.1% from 245 to 213 participants.

### 2.5 Frailty assessment

Variables for evaluating frailty were selected from the Fried frailty index and supplemented with additional indicators acting as proxies for frailty, thereby expanding the coverage of the Fried frailty test. Fried's criteria for frailty encompassed the following variables: self‐reported unintentional weight loss within the previous year, low physical activity estimated by metabolic equivalent task (MET) score and sedentary status, gait slowness assessed with the timed up‐and‐go (TUG) test, weakness evaluated through hand grip strength (HGS), and exhaustion derived from responses to the DASS‐21 and CASP‐19 questionnaires. Additional assessments to Fried's criteria included Katz activities of daily living (ADL) to evaluate independence, the Montreal cognitive assessment (MoCA) test for cognitive performance, gait speed, and the functional reach (FR) assessment as a gauge of balance and self‐reported falls in the previous year. Variables were dichotomized by recognized clinical thresholds indicative of subpar performance or by the highest/lowest population quartile, stratified by sex. Details of the assessment metric and classification criteria for each variable are included in Table 1.

**TABLE 1:** Frailty indicators selected for assessment between the postural blood pressure clusters.

Parameter	Assessment	Assessment metric	Classification criteria
Fried frailty index^(a–e)^	Sum of assessment scores	Fried frailty criteria: weight loss^(a)^, low physical activity^(b1 or b2)^, slowness^(c)^, weakness^(d)^, exhaustion^(e)^	Pre‐frail or frail phenotype: ≥2 indicators^(a–e)^
Weight loss^(a)^(unintentional)	Self‐reported	Question: ‘In the past year have you lost 10 pounds (4.5 kg) or more in weight when you weren't trying to, for example, because of illness?’	Positive response to weight loss of ≥4.5 kg within previous 12 months
Physical activity^(b1, b2)^	IPAQ (international physical activity questionnaire)	^(b1)^Total weekly MET expenditure^*^ ^(b2)^Sedentary assessment: weekly sitting hours, minutes	Lowest^(b1)^/highest^(b2)^ quintile (stratified by sex)
Speed and mobility^(c)^	TUG (timed up‐and‐go)	Time (in seconds) to rise from seated position, walk 3 m and return to seated	Reduced speed and mobility: >13.5 s
Weakness^(d)^	HGS (hand grip strength)	Jamar digital dynamometer. Mean of three measurements (in kilograms) using dominant hand	Lowest quintile (stratified by sex)
Exhaustion^(e)^	DASS‐21 (depression, anxiety and stress) CASP‐19 (control, autonomy, self‐realization and pleasure)	Statement: ‘I found it difficult to work up the initiative to do things’ (DASS‐21) Statement: ‘I feel full of energy these days’ (CASP‐19)	Positive response to DASS‐21 question and/or Negative response to CASP‐19 question
Independence	Katz ADL (activities of daily living)	Seven questions encompassing bathing, dressing, toileting, transferring, feeding and continence	Moderate loss of independence: ≤5
Cognitive performance	MoCA (Montreal cognitive assessment)	Assessment of executive function, phonemic fluency, verbal abstraction, attention, calculation, working memory and language	Cognitive performance impaired: <26
Gait	Frailty 15 ft	Walking speed (m/s) = 4.75 m (15 ft)/time (s)	Slow gait: ≤0.6 m/s
Balance	FR (functional reach)	Maximum standing reach measured at third distal interphalangeal joint (best out of two)	Balance impaired: <15 cm
Recent fall	Fall in the past 12 months	Details of fall and post‐fall follow‐up Slip/trip, loss of consciousness or non‐accidental (unexplained)	Positive response: ≥1 self‐reported within previous 12 months

*Note*: Assessments of cognitive performance, frailty and falls risk.

* Total MET (metabolic equivalent task) expenditure = (3.3 × walking min/day × walking days/week) + (4.0 × moderate activity min/day × moderate activity days/week) + (8.0 × vigorous activity min/day × vigorous activity days/week).

### 2.6 Statistical analyses

Statistical analyses were conducted using the statistics libraries available in the Python Programming Language (v.3.8.10, RRID:SCR_008394). Specific statistics libraries included SciPy (RRID:SCR_008058) and statsmodels (RRID:SCR_016074). Cluster visualizations were created using matplotlib (RRID:SCR_008624) and Plotly Python Graphing Library (RRID:SCR_013991). The normality of continuous variables was assessed with the one‐sample Kolmogorov–Smirnov test. The threshold for statistical significance was set at *P* < 0.05, except where noted as *P* < 0.017, to account for multiple comparisons using the Bonferroni correction.

Baseline characteristics of the study participants, categorized by cluster, were presented as proportional percentages for categorical variables and as means ± SD or medians with interquartile range for continuous variables. Overall cluster comparisons were made using the χ^2^ test for categorical data and the Kruskal–Wallis test for continuous data. *Post hoc* pairwise analysis of the clusters was conducted using Student's *t*‐tests for continuous data variables and χ^2^ tests for categorical data variables. Haemodynamic characteristics of the clusters were reported as the mean ± SD, with overall comparisons using ANOVA or Kruskal–Wallis tests as appropriate.

A logistic regression analysis determined the odds ratio (OR) with 95% confidence interval (CI) for cluster membership as a predictor of frailty. Confounders were chosen a priori, guided by their anticipated influence in modifying the relationship between frailty and haemodynamic stability, and their role in poorer performance of the active stand test. The Wald test assessed differences in frailty between clusters and the reference group, with three logistic regression models applied: (1) unadjusted; (2) adjusted for age and sex; (3) fully adjusted for age, sex, body mass index, resting SBP, cardiovascular disease (angina, myocardial infarction, hypertension, arrhythmia or heart failure), diabetes mellitus, antihypertensive use, polypharmacy (five or more medications, excluding health supplements) and elevated high‐sensitivity C‐reactive protein (CRP; >3 mg/L).

## 3 RESULTS

Of the 1135 beat‐to‐beat cardiovascular recordings collected in the first wave of the MELoR study, 861 met the rigorous inclusion criteria of the custom‐designed signal quality framework. From this cleaned dataset, complete data on all variables relevant to this study were available for 853 participants.

### 3.1 Clustering outcomes

The clustering model categorized beat‐to‐beat cardiovascular data of participants, based on features derived from early phase Δ% SBP to nadir (0– 15 s), stabilization point Δ% SBP (40 s) and late phase Δ% SBP to nadir (60–120 s) criteria, into three clusters (as set by *k* = 3). We introduced three terms as cluster titles to summarize the morphologies observed in each cluster. Note that the cluster names and their descriptive morphologies, outlined below, illustrate the dynamic patterns in beat‐to‐beat postural blood pressure. These terms are akin to, but extend beyond, traditional diagnostic definitions of dysregulated postural blood pressure conditions.

**Table:** 

Cluster name	Descriptive morphology	Akin to:
iOHYPO	Initial deficit	Initial orthostatic hypotension
OHYPO	Sustained deficit	Classic orthostatic hypotension
OHYPER	Persistent rise	Orthostatic hypertension

**FIGURE 2:**
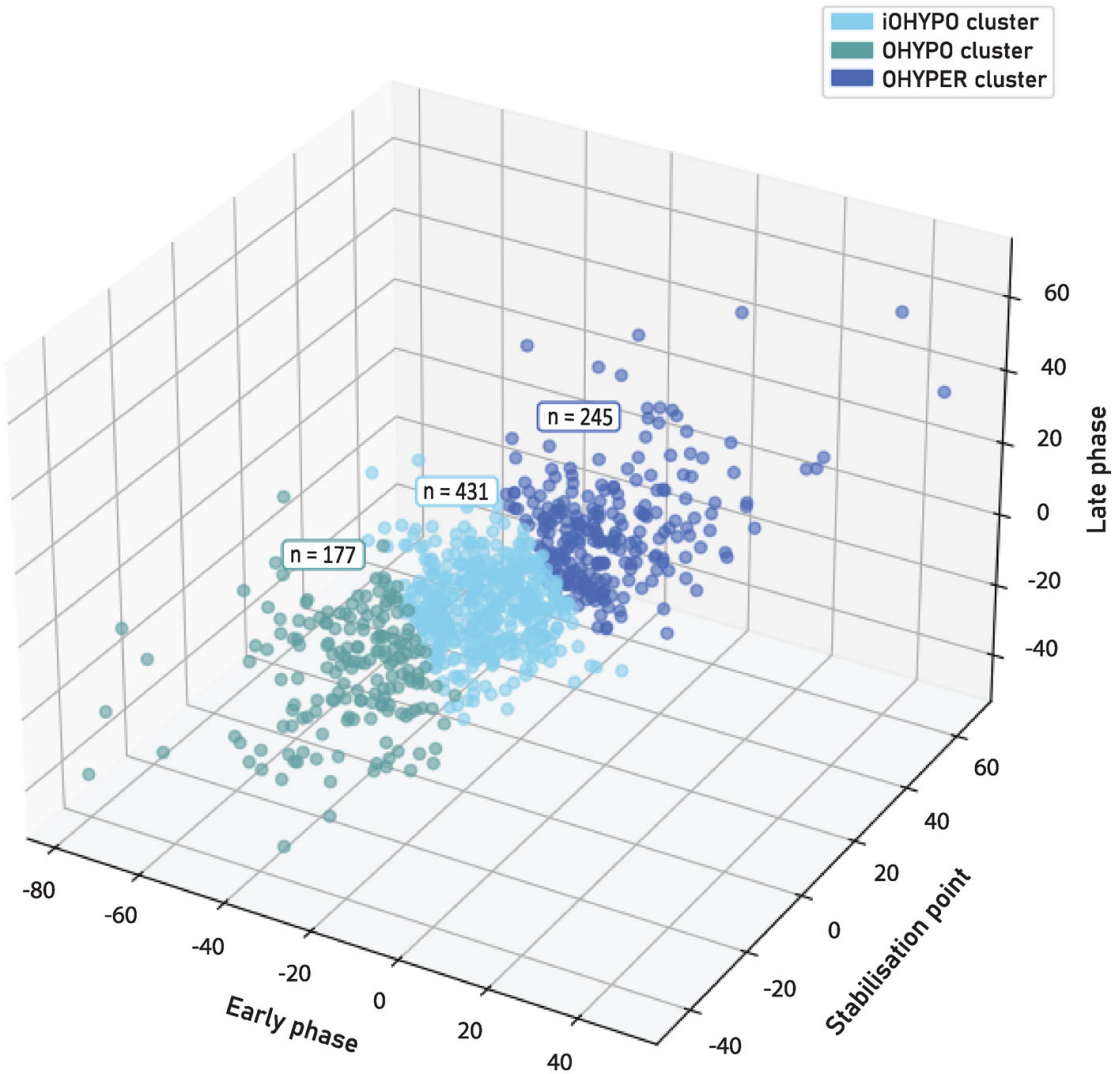
Visualization of clusters generated by *k*‐means++ clustering (*k* = 3). Assignment of participants to clusters: iOHYPO (initial deficit), OHYPO (sustained deficit) and OHYPER (persistent rise), with respective sizes (*n*).

The distribution of the clusters is depicted in the three‐dimensional representation in Figure 2. The sizes of each cluster [*n* (% of the total sample)] were as follows: iOHYPO cluster, 431 (50.5%); OHYPO cluster, 177 (20.8%); and OHYPER cluster, 245 (28.7%).

### 3.2 Study participant characteristics

Descriptive statistics for the baseline characteristics and health assessments of the sample population, grouped by cluster, are displayed in Table 2. Mean age was 68.1 ± 7.2 years, and 54% were female. Between‐cluster analysis of baseline characteristics found significant differences in relationship to sex, body mass index, supine hypertension, transient ischaemic attack (TIA), urea, CRP, use of β‐blockers, frailty (Fried frailty index), cognitive performance (MoCA), gait speed, functional mobility (TUG), hand grip strength (HGS), independence (ADL), balance (FR) and incontinence (Table 2). Subsequent *post hoc* analyses, conducted for these statistically significant findings (Table 3), identified differences between cluster pairs after applying the Bonferroni correction (*P* < 0.017). Comparisons of the iOHYPO and OHYPO clusters with the OHYPER cluster reported stronger signals of statistical significance, suggesting more pronounced deviations in participant characteristics and measured outcomes in the OHYPER cluster.

**TABLE 2:** Study participant characteristics in the total sample stratified by cluster.

Characteristic	Total sample [853 (100%)]	iOHYPO cluster [431 (50.5%)]	OHYPO cluster [177 (20.8%)]	OHYPER cluster [245 (28.7%)]	*P‐*value
Age, years, mean ± SD	68.1 ± 7.2	68.0 ± 7.2	68.5 ± 7.1	68.0 ± 7.3	0.664
Sex female, *n* (%)	458 (53.7)	217 (50.6)	78 (44.6)	161 (65.7)	**<0.001** ^§^
BMI, kg/m^2^, median [IQR]	25.0 [5.45]	24.9 [5.2]	24.5 [5.5]	25.9 [5.8]	**<0.001** ^¥^
Ethnicity					0.373
Malay, *n* (%)	302 (35.4)	157 (36.4)	57 (32.2)	88 (35.92)	
Chinese, *n* (%)	271 (31.8)	137 (31.8)	63 (35.6)	71 (29.0)	
Indian, *n* (%)	276 (32.4)	133 (30.9)	57 (32.2)	86 (35.1)	
Other, *n* (%)	4 (0.5)	4 (0.9)	0 (0)	0 (0)	
Cardiovascular disease					
Angina, *n* (%)	39 (4.6)	21 (4.9)	8 (4.5)	10 (4.1)	0.893
Myocardial infarction, *n* (%)	68 (8.0)	37 (8.6)	16 (9.0)	15 (6.1)	0.441
Hypertension, *n* (%)	449 (52.6)	219 (50.8)	88 (49.7)	142 (58)	0.138
Supine hypertension >140 mmHg, *n* (%)	95 (11.1)	64 (14.9)	17 (19.6)	14 (5.7)	**<0.001** ^§^
Arrhythmia, *n* (%)	33 (3.9)	22 (5.1)	7 (4.0)	4 (1.6)	0.079
Heart failure, *n* (%)	11 (1.3)	2 (0.5)	4 (2.3)	5 (2.0)	0.095
≥1 cardiovascular disease, *n* (%)	571 (66.9)	286 (66.4)	118 (66.7)	167 (68.2)	0.888
Musculoskeletal disease					
Arthritis (osteo/rheumatoid), *n* (%)	139 (16.3)	67 (15.6)	28 (15.8)	44 (18.0)	0.703
Osteoporosis, *n* (%)	73 (8.6)	33 (7.7)	12 (6.8)	28 (11.4)	0.154
Gout, *n* (%)	43 (5.0)	21 (4.9)	8 (4.5)	14 (5.7)	0.836
≥1 musculoskeletal disease, *n* (%)	212 (24.9)	103 (23.9)	40 (22.6)	69 (28.2)	0.345
Other					
TIA, *n* (%)	25 (2.9)	11 (2.6)	1 (0.6)	13 (5.2)	**0.014** ^§^
Diabetes, *n* (%)	245 (28.7)	120 (27.8)	54 (30.5)	71 (29.0)	0.800
Chronic kidney disease, *n* (%)	20 (2.3)	12 (2.8)	5 (2.8)	3 (1.2)	0.390
Markers of kidney function					
eGFR <60 mL/min/1.73 m^2^, *n* (%)	90 (10.6)	46 (10.7)	26 (14.7)	18 (7.4)	0.053
Urea >10 mmol/L, *n* (%)	14 (1.6)	5 (1.2)	8 (4.5)	1 (0.4)	**0.002** ^§^
Creatinine >130 µmol/L, *n* (%)	280 (32.8)	138 (32.0)	51 (28.8)	90 (36.7)	0.210
Markers of cardiovascular risk					
hsCRP >3 mg/L, *n* (%)	298 (34.9)	151 (35.0)	46 (26.0)	101 (41.2)	**0.005** ^§^
Total cholesterol:HDL ratio > 5, *n* (%)	98 (11.5)	49 (11.4)	23 (13.0)	26 (10.6)	0.746
Medication					
Vasodilators/nitrates, *n* (%)	23 (2.7)	11 (2.6)	5 (2.8)	7 (2.9)	0.966
α‐Blockers, *n* (%)	26 (3.1)	14 (3.3)	8 (4.5)	4 (1.6)	0.221
β‐Blockers, *n* (%)	140 (16.4)	78 (18.1)	18 (10.2)	44 (18.0)	**0.042** ^§^
Calcium channel blockers, *n* (%)	222 (26.0)	104 (24.1)	47 (26.6)	71 (29.0)	0.379
c, *n* (%)	218 (25.6)	103 (23.9)	47 (26.6)	68 (27.8)	0.512
Diuretics, *n* (%)	87 (10.2)	38 (8.8)	17 (9.6)	32 (13.1)	0.206
Antihypertensives					
≥1, *n* (%)	416 (48.8)	198 (45.9)	87 (49.2)	131 (53.5)	0.169
≥3, *n* (%)	206 (24.2)	102 (23.8)	44 (24.9)	60 (24.6)	0.289
Psychoactive medications, *n* (%)	89 (10.4)	45 (10.4)	15 (8.5)	29 (11.8)	0.537
Polypharmacy ≥5, *n* (%)	274 (32.1)	134 (31.1)	65 (36.7)	75 (30.6)	0.335
Functional, frailty and cognitive assessment					
Fried frailty ≥2 indicators^(a–e)^	225 (26.4)	107 (24.8)	35 (19.8)	83 (33.9)	**0.003** ^§^
^a^ Weight loss >4.5 kg, *n* (%)	57 (6.7)	26 (6.0)	12 (6.8)	19 (7.8)	0.688
Psych. DASS‐21, mean ± SD	5.4 ± 6.2	5.7 ± 6.4	5.6 ± 6.7	4.9 ± 5.3	0.688
Psych. CASP‐19, mean ± SD	27.9 ± 5.5	27.6 ± 5.5	28.6 ± 4.9	27.8 ± 5.8	0.131
Cognition MoCA, mean ± SD	22 ± 5.1	22.2 ± 5.2	22.6 ± 4.4	21.1 ± 5.4	**0.004** ^¥^
Physical activity IPAQ, mean ± SD	39.5 ± 73.1	41.3 ± 81.8	42.8 ± 70.0	33.9 ± 57.1	0.147
^b1^ Low physical activity, *n* (%)	175 (20.5)	82 (19.0)	34 (19.2)	59 (24.1)	0.261
^b2^ Sedentary, *n* (%)	248 (29.1)	122 (28.3)	48 (27.1)	78 (31.8)	0.507
Gait speed, m/s, mean ± SD	0.79 ± 0.19	0.81 ± 0.19	0.82 ± 0.19	0.75 ± 0.19	**<0.001** ^¥^
^c^ Mobility TUG slow >13.5 s, *n* (%)	231 (27.1)	101 (23.4)	44 (24.9)	86 (35.1)	**0.003** ^§^
Strength HGS, kg, mean ± SD	23.6 ± 8.0	23.7 ± 8.1	24.2 ± 7.5	22.3 ± 8.3	**<0.001** ^¥^
^d^ HGS low, sex‐adjusted quintile, *n* (%)	171 (20.1)	82 (19.0)	29 (16.4)	60 (24.5)	0.092
Independence ADL, mean ± SD	5.7 ± 0.6	5.7 ± 0.5	5.8 ± 0.4	5.6 ± 0.8	**0.018** ^¥^
Balance FR, cm, mean ± SD	25.7 ± 7.2	26.2 ± 7.1	26.6 ± 7.4	24.3 ± 7.0	**0.002** ^¥^
Incontinence, *n* (%)	229 (27.0)	99 (23.0)	32 (18.1)	72 (29.4)	**0.023** ^§^
^e^ Exhaustion, *n* (%)	90 (10.6)	46 (10.7)	21 (11.9)	23 (9.4)	0.711
Fall (≥1 within previous 12 months)	181 (21.2)	36 (17.2)	33 (18.1)	48 (22.5)	0.410

*Note*: Study participant characteristics for the total sample and per cluster: iOHYPO (initial deficit), OHYPO (sustained deficit) and OHYPER (persistent rise). Data are presented as *n* (%), mean ± SD or median [IQR: quartile 3–quartile 1]. *P‐*value: overall comparison of variable across all clusters.

Abbreviations: ACEi/ARB, angiotensin‐converting enzyme inhibitor/angiotensin II receptor blocker; ADL, activities of daily living; BMI, body mass index; CASP‐19, control, autonomy, self‐realization and pleasure; DASS‐21, depression, anxiety and stress; FR, functional reach; HDL, high‐density lipoprotein; HGS, hand grip strength; hsCRP, high‐sensitivity C‐reactive protein; IPAQ, international physical activity questionnaire; IQR, interquartile range; MoCA, Montreal cognitive assessment; SBP, systolic blood pressure; TIA, transient ischaemic attack; TUG, timed up‐and‐go.

Continuous data variables (all non‐parametric): ^¥^Kruskal–Wallis.

Categorical data variables: ^§^χ^2^. Statistically significant *P‐*values (*P* < 0.05) are in bold.

**TABLE 3:** *Post hoc* analysis of participant characteristics for pairwise cluster comparisons.

Characteristic	iOHYPO cluster vs. OHYPO cluster		iOHYPO cluster vs. OHYPER cluster		OHYPO cluster vs. OHYPER cluster	
		*P*‐value		*P*‐value		*P*‐value
Sex (female)	0.06	0.186	−0.15	**<0.001**	−0.21	**<0.001**
BMI (kg/m^2^)	0.70 ± 0.39	0.072	−0.99 ± 0.39	**0.011**	−1.70 ± 0.45	**<0.001**
Supine hypertension	0.05	0.084	0.09	**<0.001**	0.04	0.131
TIA	0.02	0.109	−0.03	0.063	−0.05	**0.007**
Urea >10 mmol/L	−0.03	**0.009**	0.01	0.316	0.04	**0.004**
hsCRP >3 mg/L	0.09	0.030	−0.06	0.110	−0.15	**0.001**
β‐Blockers	0.08	**0.015**	0.01	0.964	−0.08	0.026
FFI indicators ≥2	0.05	0.181	−0.09	**0.012**	−0.14	**0.001**
Cognition, MoCA	−0.40 ± 0.41	0.333	1.15 ± 0.43	**0.007**	1.55 ± 0.47	**0.001**
Gait speed (m/s)	−0.01 ± 0.02	0.585	0.06 ± 0.02	**<0.001**	0.07 ± 0.02	**<0.001**
Mobility, TUG >13.5 s	−0.01	0.708	−0.12	**0.001**	−0.10	0.025
Strength, HGS (kg)	−0.51 ± 0.68	0.996	1.43 ± 0.65	**0.003**	1.92 ± 0.77	**0.013**
Independence, ADL	−0.07 ± 0.04	0.084	0.12 ± 0.05	0.035	0.18 ± 0.06	**0.001**
Balance, FR (cm)	−0.38 ± 0.65	0.563	1.87 ± 0.56	**0.001**	2.24 ± 0.71	**0.002**
Incontinence	0.05	0.183	−0.06	0.065	−0.11	**0.008**

*Note*: Post hoc analysis of statistically significant variables from the study participant characteristics (Table 2). Comparison between paired clusters: iOHYPO (initial deficit), OHYPO (sustained deficit) and OHYPER (persistent rise). Differences are reported as the mean ± SD for continuous data and as proportional values for categorical data. Pairwise analysis was conducted using Student's *t*‐tests for continuous data and χ^2^ tests for categorical data. Statistically significant *P‐*values with Bonferroni correction (*P* < 0.017) are in bold.

Abbreviations: ADL, activities of daily living; BMI, body mass index; FR, functional reach; HGS, hand grip strength; hsCRP, high‐sensitivity C‐reactive protein; MoCA, Montreal cognitive assessment; TIA, transient ischaemic attack; TUG, timed up‐and‐go.

### 3.3 Haemodynamic characteristics

**FIGURE 3:**
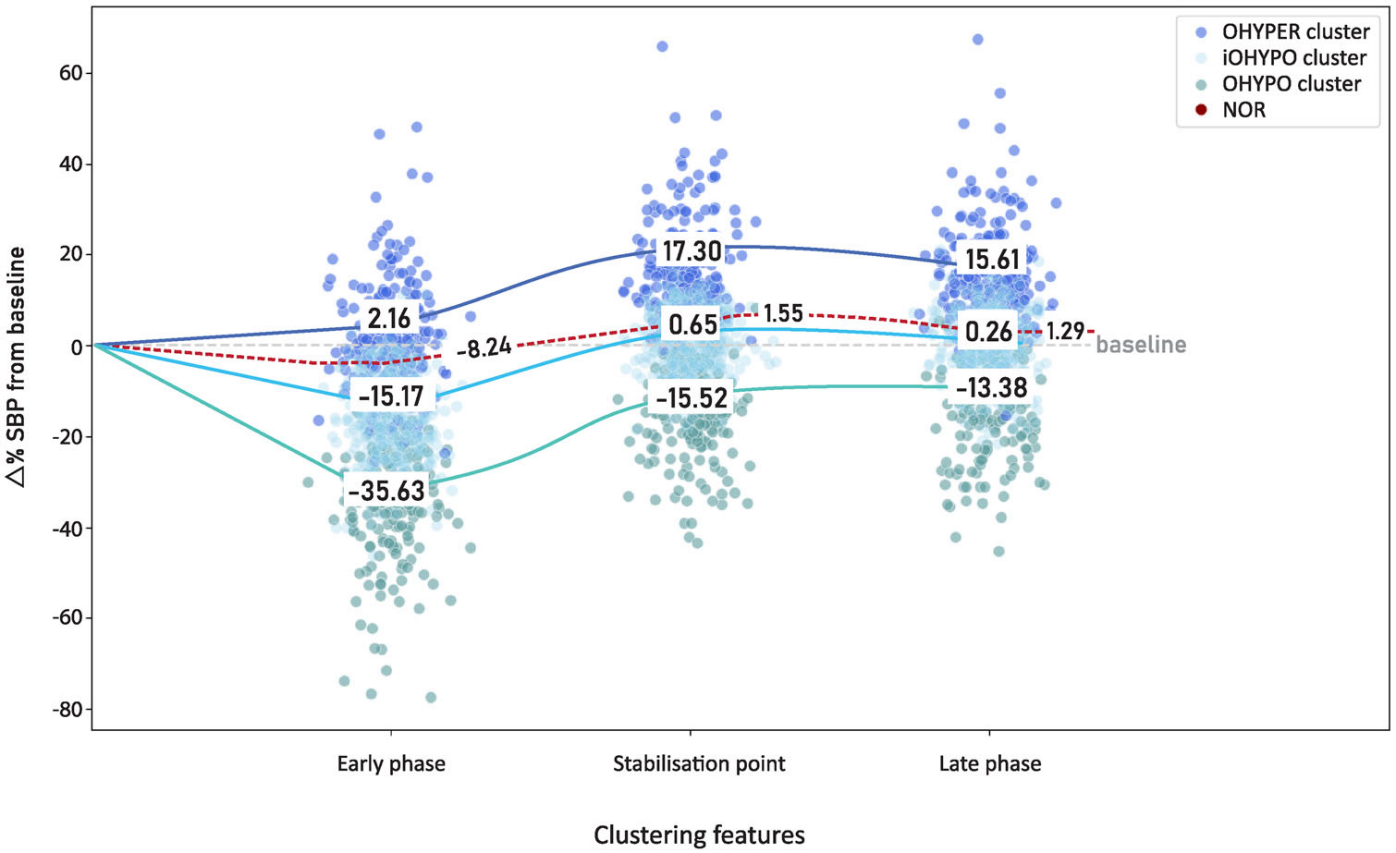
Trend analysis of postural blood pressure profiles in the clusters: OHYPER (persistent rise), iOHYPO (initial deficit), OHYPO (sustained deficit) and NOR (normal orthostatic response) group, with associated trend lines. Distribution of features in early phase, stabilization point and late phase, with mean magnitude of shift from baseline (Δ% SBP). Reciprocal NOR mean values for each feature are included along the red dashed line. Abbreviation: SBP, systolic blood pressure.

The mean values of the clustering feature variables (early phase, stabilization point and late phase) for each cluster are depicted in Figure 3. This illustration reveals distinct trends, providing insight into the orthostatic SBP profiles of individuals within each cluster. Unsurprisingly, the OHYPER cluster (persistent rise) displayed consistently elevated SBP at all feature points, with positive percentage shifts from baseline (2.16%, 17.30% and 15.61%). The iOHYPO cluster (initial deficit) showed a marked early phase drop in SBP (−15.7%), thereafter recovering to baseline levels (0.65% and 0.26%). The OHYPO cluster (sustained deficit) displayed a continued SBP deficit from baseline (−35.63%, −15.52% and −13.38%) at all three clustering feature points, failing to return to baseline throughout the 2 min standing phase of the active stand test. After a transient deviation from the baseline at the early phase, the NOR reference group exhibited a trend that recovered and remained close to the baseline at the feature points (−8.24%, 1.55% and 1.29%).

Resting cardiovascular indices derived from the supine baseline phase of the active stand test and an analysis of SBP shifts from baseline (expressed as the magnitude as a percentage and in millimetres of mercury) at the three feature points for the clusters, and for the normal orthostatic response (NOR) reference group, are presented in Table 4. Analyses indicated statistically significant variation (*P* < 0.05) across clusters for SBP, DBP and mean arterial pressure (MAP). Pulse pressure (PP), heart rate (HR) and the RR 30:15 ratio (a marker of parasympathetic heart rate control) did not reach significance (*P* > 0.05). As anticipated, all features used to build the clusters (magnitude of shift from baseline as a percentage), in addition to the values (in millimetres of mercury) at these points, were significantly different across the clusters (*P* < 0.001).

**TABLE 4:** Haemodynamic characteristics of the total sample, normal orthostatic response group and clusters.

Characteristic	Total sample [853 (100%)]	NOR reference group [254 (29.8%)]	iOHYPO cluster[431 (50.5%)]	OHYPO cluster [177 (20.8%)]	OHYPER cluster [245 (28.7%)]	*P*‐value
Resting index						
SBP, mmHg	116.1 ± 19.4	121.4 ± 19.6	119.1 ± 18.7	115.6 ± 18.9	111.2 ± 20.1	<0.001^‡^
DBP, mmHg	70.4 ± 14.3	74.2 ± 14.6	72.6 ± 14.0	68.8 ± 13.9	67.6 ± 14.5	<0.001^‡^
PP, mmHg	45.7 ± 12.0	47.2 ± 12.2	46.5 ± 11.6	46.8 ± 14.1	43.6 ± 10.7	0.094^‡^
MAP, mmHg	85.6 ± 15.2	89.8 ± 15.4	88.1 ± 14.7	84.4 ± 14.2	82.1 ± 15.8	<0.001^†^
HR, beats/min	70.2 ± 11.0	70.7 ± 10.4	70.3 ± 10.6	69.2 ± 11.2	70.6 ± 11.5	0.606^†^
RR, 30:15 ratio	1.16 ± 0.15	1.16 ± 0.15	1.16 ± 0.16	1.15 ± 0.13	1.15 ± 0.15	0.266^†^
Clustering feature						
Early phase, Δ%	−14.4 ± 16.8	−8.2 ± 7.4	−15.2 ± 9.3	−35.6 ± 12.2	2.2 ± 11.3	<0.001^‡^
Stabilization point, Δ%	2.1 ± 14.1	1.6 ± 5.1	0.7 ± 6.6	−15.5 ± 9.5	17.3 ± 9.6	<0.001^‡^
Late phase, Δ%	1.8 ± 13.6	1.3 ± 5.3	0.3 ± 7.3	−13.4 ± 10.8	15.6 ± 10.4	<0.001^‡^
Early phase, ΔmmHg	−16.8 ± 19.5	−9.9 ± 8.9	−17.9 ± 11.1	−41.0 ± 15.5	2.4 ± 11.8	<0.001^‡^
Stabilization point, ΔmmHg	1.9 ± 15.9	1.8 ± 6.4	0.7 ± 7.8	−18.3 ± 12.8	18.5 ± 9.0	<0.001^‡^
Late phase, ΔmmHg	1.6 ± 15.3	1.5 ± 6.5	0.2 ± 8.7	−15.8 ± 13.8	16.6 ± 10.1	<0.001^‡^

*Note*: Haemodynamic parameters obtained from beat‐to‐beat cardiovascular assessment in the total sample, normal orthostatic response (NOR) reference group and per cluster: iOHYPO (initial deficit), OHYPO (sustained deficit) and OHYPER (persistent rise). Data are presented as the mean ± SD. Resting cardiovascular indices are taken from the supine baseline time range (60–30 s prior to standing upright). Clustering features are displayed as the magnitude of shift from baseline as a percentage and in millimetres of mercury.

Abbreviations: DBP, diastolic blood pressure, HR, heart rate; MAP, mean arterial pressure; PP, pulse pressure as an indicator of stroke volume; RR 30:15 ratio, interval duration difference between heartbeats at 15 and 30 s as an indicator of heart rate recovery; SBP, systolic blood pressure.

^†^ Kruskal–Wallis.

^‡^ ANOVA.

Statistical significance: *P* < 0.05.

### 3.4 Cluster distribution and quality metrics

Table 5 presents cluster metrics for the model using *k* = 3, listing the feature vector average distances of participants from cluster centroids (*d*_c_) ranging between 12.48 and 16.65 Δ% SBP from baseline. Cluster density, gauged by cluster diameters (ᴓ), ranged from 38.16 to 75.76 Δ% SBP from baseline. A pairwise analysis of the distances between cluster centroids (*d*_cc_) of all the clusters is included. Average distances to each cluster centroid varied across the three variables chosen to cluster the data, with the Δ% SBP from baseline in the early phase (*d*_c_ *E*_(0–15)_) ranging from 7.56 to 9.23, the 40 s stabilization point (*d*_c_ *S*_(40)_) ranging from 5.32 to 7.36, and the late phase (*d*_c_ *L*_(5–120)_) ranging from 5.78 to 8.61.

**TABLE 5:** Cluster distribution and quality metrics.

Cluster *k* = 3	*d*_c_	ᴓ	*d*_cc_	*d*_c_ *E*_(0–15)_	*d*_c_ *S*_(40)_	*d*_c_ *L*_(60–120)_
iOHYPO cluster^1^	15.41	75.76		8.45	7.24	7.75
OHYPO cluster^2^	16.65	55.90		9.23	7.36	8.61
OHYPER cluster^3^	12.48	38.16		7.56	5.32	5.78
			^1^ versus ^3^ 28.56			
			^2^ versus ^3^ 29.50			
			^2^ versus ^1^ 57.95			

Additional metrics	*k* = 2	*k* = 3	*k* = 4	*k* = 5	*k* = 6	
*J*	0.50	0.33	0.25	0.20	0.17	
*I*_conv_	7	8	26	32	18	
*S*_sil_	0.39	0.33	0.29	0.28	0.27	

*Note*: Cluster quality metrics for the *k*‐means++ model for each cluster: iOHYPO (initial deficit), OHYPO (sustained deficit) and OHYPER (persistent rise). Abbreviations: *d*_c_, average distance to cluster centre; ᴓ, diameter, maximum distance to cluster centre; *d*_cc_, distances between cluster centres; *d*_c_ *E*_(0–15)_, early phase feature, average distance to cluster centre; *d*_c_ *S*_(40)_, stabilization point feature, average distance to cluster centre; *d*_c_ *L*_(60–120)_, late phase feature, average distance to cluster centre; *I*_conv_, number of iterations before convergence; *J*, average Jaccard similarity across bootstraps; *S*_sil_, silhouette score for *k* = *x*, chosen value of *k* for cluster model shaded.

The additional metrics in Table 5, summarizing the clustering model outputs that informed the optimal *k* selection, indicate that the highest quality was achieved with *k* = 2, followed by *k *= 3. This is reflected in the Jaccard similarity coefficients (0.50 for *k* = 2 and 0.33 for *k* = 3), the number of iterations to convergence (seven for *k* = 2 and eight for *k* = 3) and the silhouette scores (0.39 for *k* = 2 and 0.33 for *k* = 3).

### 3.5 Frailty assessment

Compared with the NOR reference group, participants in orthostatic hypotensive clusters, iOHYPO and OHYPO, reported the most favourable frailty indicators. Conversely, the orthostatic hypertensive cluster, OHYPER, demonstrated the poorest performance in the assessed frailty indicators.

When examining specifically the fully adjusted logistic regression model, statistically significant outcomes (*P* < 0.05) emerged in three areas: the iOHYPO cluster showed better cognitive performance [OR, 0.51; 95% CI: 0.33; 0.79], the OHYPO cluster was less frail by Fried's frailty index [OR, 0.60; 95% CI: 0.36; 0.98], and the OHYPER cluster exhibited slower gait speed [OR, 1.74; 95% CI: 1.01; 2.99].

In the iOHYPO cluster, participants exhibited superior cognitive performance in all logistic regression models (unadjusted, age and sex adjusted, and fully adjusted), with *P*‐values of 0.001, 0.001 and 0.002, respectively. There was also a lower likelihood of having experienced falls in the past year in the unadjusted and age‐sex adjusted models (*P* = 0.039 and *P* = 0.043). However, this significance was no longer present after controlling for confounders in the fully adjusted model.

In the OHYPO cluster, participants retained independence in daily activities in the unadjusted model (*P* = 0.047) and were less frail according to the Fried frailty index in both age and sex‐adjusted (*P* = 0.020) and fully adjusted models (*P* = 0.042).

In the OHYPER cluster, participants had slower gait speeds which persisted through all logistic regression models: unadjusted (*P* = 0.005); age and sex adjusted (*P* = 0.017); and fully adjusted (*P* = 0.044). Additionally, significance was found with reduced functional reach indicative of impaired balance, in both unadjusted (*P* = 0.026) and age‐ and sex‐adjusted (*P* = 0.049) models.

**FIGURE 4:**
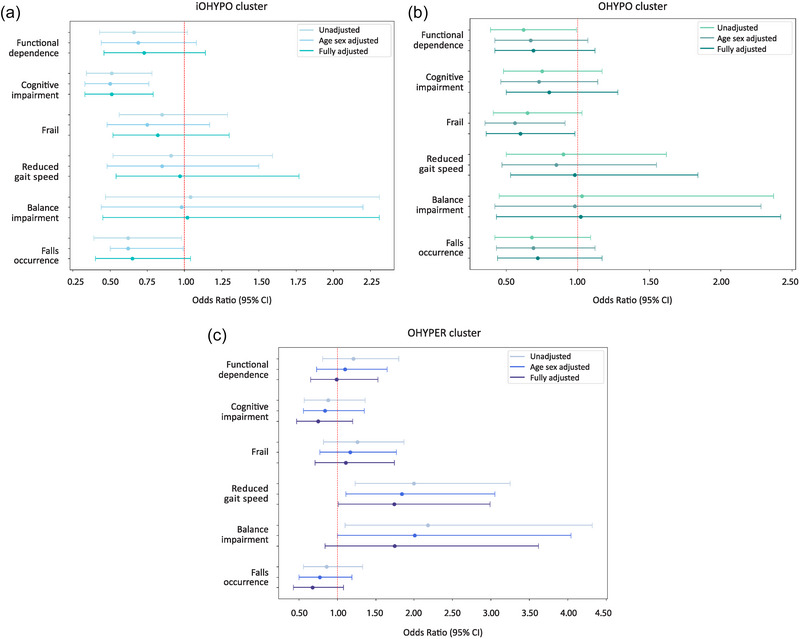
Odds ratios (depicted as points) and 95% confidence intervals (CI; represented by whiskers) for clinical assessments in clusters iOHYPO (initial deficit; a), OHYPO (sustained deficit; b) and OHYPER (persistent rise; b), relative to the normal orthostatic response (NOR) reference group, across three logistic regression models: unadjusted, age and sex adjusted, and fully adjusted. Red dashed line represents odds below one indicating lower likelihood and above one indicating higher likelihood of the evaluated clinical variable in each cluster (a–c). Models where horizontal whiskers do not intersect with the red dashed line indicate statistical significance (*P* < 0.05).

The complete analysis is presented in Table 6, accompanied by visual representations of the ORs and CIs for all frailty indicators in forest plots shown in Figure 4.

**TABLE 6:** Comparative analysis of frailty across clusters versus normal orthostatic response reference group.

	Models [*n* (%)]	iOHYPO cluster [209 (24.5)]		OHYPO cluster [177 (20.8)]		OHYPER cluster [213 (25.0)]		NOR (reference) [254 (29.8)]
Independence compromised, ADL score ≤ 5	*n* (%)	42 (20.1)		34 (19.2)		67 (31.5)		70 (27.6)
		OR (95% CI)	*P*‐value	OR (95% CI)	*P*‐value	OR (95% CI)	*P*‐value	
	Unadj.	0.66 (0.43, 1.02)	0.063	0.62 (0.39, 0.99)	**0.047**	1.21 (0.81, 1.80)	0.357	
	Adj. age, sex	0.69 (0.44, 1.08)	0.108	0.67 (0.42, 1.07)	0.095	1.10 (0.73, 1.65)	0.657	
	Fully adj.	0.73 (0.46, 1.14)	0.166	0.69 (0.42, 1.12)	0.131	0.99 (0.65, 1.53)	0.981	
Cognitive performance impaired, MoCA score <26	*n* (%)	137 (65.6)		130 (73.5)		162 (76.1)		201 (79.1)
		OR (95% CI)	*P*‐value	OR (95% CI)	*P*‐value	OR (95% CI)	*P*‐value	
	Unadj.	0.51 (0.34, 0.78)	**0.001**	0.75 (0.48, 1.17)	0.203	0.88 (0.57, 1.36)	0.567	
	Adj. age, sex	0.50 (0.33, 0.76)	**0.001**	0.73 (0.46, 1.14)	0.164	0.87 (0.56, 1.35)	0.525	
	Fully adj.	0.51 (0.33, 0.79)	**0.002**	0.80 (0.50, 1.28)	0.352	0.75 (0.47, 1.20)	0.228	
Frail, Fried frailty phenotype, ≥2 indicators	*n* (%)	51 (24.4)		35 (19.8)		69 (32.4)		70 (27.6)
		OR (95% CI)	*P*‐value	OR (95% CI)	*P*‐value	OR (95% CI)	*P*‐value	
	Unadj.	0.85 (0.56, 1.29)	0.442	0.65 (0.41, 1.03)	0.065	1.26 (0.82, 1.87)	0.255	
	Adj. age, sex	0.75 (0.48, 1.17)	0.200	0.56 (0.35, 0.91)	**0.020**	1.17 (0.77, 1.77)	0.474	
	Fully adj.	0.82 (0.52, 1.30)	0.408	0.60 (0.36, 0.98)	**0.042**	1.11 (0.71, 1.74)	0.634	
Slow gait, speed ≤ 0.6 m/s	*n* (%)	25 (12.0)		21 (11.9)		49 (23.0)		33 (13.0)
		OR (95% CI)	*P*‐value	OR (95% CI)	*P*‐value	OR (95% CI)	*P*‐value	
	Unadj.	0.91 (0.52, 1.59)	0.739	0.90 (0.50, 1.62)	0.728	2.00 (1.23, 3.25)	**0.005**	
	Adj. age, sex	0.85 (0.48, 1.50)	0.560	0.85 (0.47, 1.55)	0.598	1.84 (1.11, 3.05)	**0.017**	
	Fully adj.	0.97 (0.54, 1.77)	0.931	0.98 (0.53, 1.84)	0.959	1.74 (1.01, 2.99)	**0.044**	
Balance impaired, functional reach of <15 cm	*n* (%)	12 (5.7)		10 (5.7)		24 (11.3)		14 (5.5)
		OR (95% CI)	*PP*‐value	OR (95% CI)	*P*‐value	OR (95% CI)	*P*‐value	
	Unadj.	1.04 (0.47, 2.31)	0.915	1.03 (0.45, 2.37)	0.915	2.18 (1.10, 4.32)	**0.026**	
	Adj. age, sex	0.98 (0.44, 2.20)	0.970	0.98 (0.42, 2.28)	0.960	2.01 (1.00, 4.04)	**0.049**	
	Fully adj.	1.02 (0.45, 2.31)	0.953	1.02 (0.43, 2.42)	0.929	1.75 (0.84, 3.62)	0.132	
Recent fall, ≥1 self‐reported within previous 12 months	*n* (%)	37 (17.7)		32 (18.1)		48 (22.5)		64 (25.2)
		OR (95% CI)	*P*‐value	OR (95% CI)	*P*‐value	OR (95% CI)	*P*‐value	
	Unadj.	0.62 (0.39, 0.98)	**0.039**	0.68 (0.42, 1.09)	0.110	0.86 (0.56, 1.33)	0.502	
	Adj. age, sex	0.62 (0.50, 0.99)	**0.043**	0.69 (0.43, 1.12)	0.136	0.77 (0.50, 1.19)	0.238	
	Fully adj.	0.65 (0.40, 1.04)	0.075	0.72 (0.44, 1.17)	0.186	0.68 (0.43, 1.08)	0.103	

*Note*: Odds ratios (OR) and 95% confidence intervals (CI) for frailty indicators across clusters, compared with the normal orthostatic response (NOR) group. Logistic regression models controlled for confounders.

Abbreviations: Adj. age, sex, adjusted for age and sex; Fully adj., adjusted for age, sex, body mass index, resting systolic blood pressure, cardiovascular disease, diabetes, antihypertensives and polypharmacy; Unadj., unadjusted model.

Statistically significant Wald *P‐*values (*P* < 0.05) are in bold.

## 4 DISCUSSION

Our study assigned individuals to clusters based on their postural blood pressure, addressing two key questions: would machine learning‐derived clusters align broadly with known patterns of orthostatic blood pressure dysregulation, and would they show differences in levels of frailty? Following the clustering process, these aims were met. First, AI identified three distinct clusters, termed iOHYPO, OHYPO and OHYPER, based on their postural blood pressure morphology, which resembled initial orthostatic hypotension, classic orthostatic hypotension and orthostatic hypertension, respectively. Second, frailty outcomes differed between them, suggesting differences between the clusters that could have clinical value.

### 4.1 Orthostatic hypertension

Individuals in our OHYPER cluster demonstrated poorer performance in several frailty assessments and one marker of inflammation. In line with the recently published findings of Choi et al. (2024), these findings suggest that individuals with orthostatic rises in blood pressure are more frail.

The pathophysiology of orthostatic hypertension is not yet understood. Similar to orthostatic hypotension, the initial gravitational redistribution of blood towards the abdomen and lower extremities upon standing is thought to be the precursor event, setting the conditions for the subsequent cascade of responses. It is hypothesized that orthostatic hypertension stems from an exaggerated compensatory mechanism to facilitate the return of this translocated blood volume back to the heart. In response to transient blood pressure drop, the baroreflex initiates a withdrawal of parasympathetic activity coupled with a gain in sympathetic provision, influencing cardiac contractility and leading to increases in heart rate and rapid adjustments in vascular tone (Borst et al., 1982; Smith et al., 1994). Once blood pressure exceeds baseline, counter‐regulatory vagal tone re‐engages with corresponding cardiovascular adjustments to stabilize the pressures. However, in the case of orthostatic hypertension, it is thought that malfunction somewhere within these regulatory mechanisms results in excessively increased postural blood pressure.

In older individuals, there is an increased dependence on the sympathetic baroreflex to preserve haemodynamic stability as the cardiovagal system ages (Jones et al., 2001). This is seen in the higher resting heart rates recorded in the OHYPER cluster. Thus, it could be theorized that orthostatic hypertension is an adaptive mechanism rather than a purely pathophysiological one. In the context of this condition, arterial stiffness caused by vascular ageing would contribute to alterations in vascular resistance and impaired arterial baroreceptors, with subsequent augmented pressor responses (Kario, 2013; Magkas et al., 2019).

Interestingly, our OHYPER cluster reported elevated serum CRP levels, which have been observed with arterial stiffness (Mattace‐Raso et al., 2004). In addition to being an indication of increased cardiovascular risk, this inflammatory marker has been independently associated with slower gait speed (Wawrzyniak‐Gramacka et al., 2021) and impaired muscle strength (Shokri‐Mashhadi et al., 2021); outcomes that were observed in our OHYPER cluster. After adjusting for CRP in our logistic regression model, the signal for significantly slower gait speed persisted in the OHYPER cluster, hence without further investigation, this frailty outcome does not appear to be mediated directly by the source of the elevated CRP. Although the underlying reason for CRP elevation cannot be determined, mildly elevated CRP is known to occur outside of the normal short‐term responses to infection and trauma in population‐based studies. It is unlikely that these factors were present in our study population, who were not acutely ill during their health check. Thus, the elevated CRP and morphology of the OHYPER cluster could be suggestive of cardiovascular risk. Low‐grade inflammation, detected through CRP, is widely recognized for its association with end‐organ damage and cardiovascular events, particularly coronary heart disease. Notably, using the same thresholds we adopted in our analyses, elevated CRP levels have been shown nearly to double the risk of coronary heart disease at 10‐year follow‐up (Cushman et al., 2005).

Transient ischaemic attacks, like silent cerebral infarcts, are adverse cerebral perfusion events. In our OHYPER cluster, we observed the highest incidence of TIA, mirroring findings from a study in which the prevalence of silent cerebral infarcts was greater in orthostatic hypertension than in orthostatic hypotension (Eguchi et al., 2004). The authors attributed the increased risk to the magnitude of change in postural blood pressure. Indeed, orthostatic hypertension has been used as a predictor of cardiovascular risk and is seen with increased blood pressure variability (Kario, 2013). This is interesting, because the individuals in our OHYPER cluster had the lowest resting SBP and were not hypertensive. Given the association with frailty, perhaps the OHYPER cluster has increased vulnerability here, aligning with the known blood pressure variability and excessive rises in systemic blood pressure observed in orthostatic hypertension.

It should be noted that the resting SBP is particularly low among MELoR study participants compared with what could be expected in an older population prone to hypertension and other cardiovascular comorbidities. The resting SBP measurements were extracted from the supine phase of the active stand test, and although lower SBP has been observed in supine positions relative to seated positions (Lacruz et al., 2017), the values in this study are notably lower than in other beat‐to‐beat studies using the same baseline period (Finucane, Savva, & Kenny, 2017; Moloney et al., 2021). However, our averages are consistent with those reported by colleagues using the same dataset (Saedon, Frith, Goh, et al., 2020) and in a cohort study of Malaysian fallers (>65 years) referred from emergency department and primary care clinics, where resting SBP measurements were only marginally higher (Goh et al., 2016). This presents an interesting haemodynamic profile within this demographic, warranting further exploration. Furthermore, the resting SBP is expected to be relevant to diagnosis of orthostatic hypertension, where a recent proposal for consensus recommends basing diagnosis not only on the magnitude of change but reserving the term ‘orthostatic hypertension’ for individuals also with a standing blood pressure of ≥140 mmHg (Jordan et al., 2023).

### 4.2 Orthostatic hypotension

Individuals in the clusters with postural blood pressure profiles indicative of initial orthostatic hypotension and classic orthostatic hypotension outperformed the NOR reference group in frailty assessments. This discovery deviates from the bulk of existing research, in which individuals experiencing drops in blood pressure upon standing tend to have poorer outcomes than those with a stable haemodynamic response. However, our results are corroborated in another study that found individuals with initial orthostatic hypotension and classic orthostatic hypotension had superior frailty metrics and cognitive performance (Saedon, Frith, Goh, et al., 2020).

The iOHYPO cluster showed better cognitive performance across all logistic regression models. Blood pressure fluctuations pose a risk of intermittent cerebral hypoperfusion and cognitive decline, noted with orthostatic hypotension in the absence of neurodegenerative disease (Sambati et al., 2014). It remains uncertain whether the short‐lived blood pressure deficit in initial orthostatic hypotension elicits chronic effects or, at minimum, temporary symptoms. The superior cognitive performance in our iOHYPO cluster suggests that a blood pressure drop in the first 15 s of standing does not appear, at least in our cohort, to be deleterious to cognition.

In managing orthostatic hypotension, immediate concerns focus on the significantly increased risk of falls and how to avoid them (Mol et al., 2019). It is noteworthy that in the OHYPO cluster, β‐blocker use was significantly lower, and more individuals were hypertensive. This is not surprising, because essential hypertension is commonly associated with orthostatic hypertension (Juraschek et al., 2023). A diagnosis of orthostatic hypotension in this group might have led to the discontinuation of β‐blockers to address the risk of falls, which is reflected in the lower prescription of this medication in the group. A significantly lower occurrence of falls was reported in the iOHYPO cluster in our study. A thorough falls investigation by Finucane, O'Connell, Donoghue, et al. (2017) found that only initial orthostatic hypotension revealed no association with falls, and that delayed recovery at 40 s and sustained orthostatic hypotension were independent predictors. Thus, although falls are expected to be less frequent in initial orthostatic hypotension than in classic orthostatic hypotension, the iOHYPO cluster's fall‐protective advantage over our normal orthostatic response reference group is not fully understood but could be related to the improved cognitive function of study participants in this cluster.

Upon standing, the bulk of blood volume shifted to the abdomen and lower limbs is contained in the splanchnic–mesenteric bed and deep intra‐ and intermuscular leg veins (Buckey et al., 1988). The skeletal muscle pump in these areas contributes to the return of blood to the central circulation by passively relaxing to allow hydrostatic pressure‐induced filling of surrounding vessels and actively contracting to empty them. In orthostatic hypotension, a drop in blood pressure as a result of reduced venous return by muscle insufficiency is a reasonable sarcopenia sequela; however, in orthostatic hypertension, the rise seen in blood pressure pertaining to this mechanism is less intuitive.

Initial orthostatic hypotension is thought to stem from a temporal mismatch between vascular resistance and cardiac output (Wieling et al., 2007). Passive postural adjustments, such as head‐up tilt, attenuate initial orthostatic hypotension, suggesting a fundamental relationship to the act of standing itself (Thomas et al., 2009). Rapid standing, with subsequent swift relocation of blood from splanchnic and limb muscle activation, supports the theory of healthy compensation by the baroreflex (Tanaka et al., 1996). Our study found that individuals in the iOHYPO cluster completed the TUG test significantly more quickly, hence this, coupled with the cluster's compensatory recovery of initial blood pressure drop, could demonstrate that older individuals with isolated initial orthostatic hypotension might exhibit a resilient cardiovascular system.

### 4.3 Prevalence of dysregulated postural blood pressure conditions

The assignment of 20.8% of study participants to our OHYPO cluster is consistent with the prevalence of orthostatic hypotension among older individuals in community settings, as reported in pooled results of a systematic review and meta‐analysis (Saedon, Pin Tan, & Frith, 2020). Orthostatic hypertension prevalence rates are suggested to be comparable to those of orthostatic hypotension but vary widely from 5% to 30% (Jordan et al., 2020). Agreement on its prevalence is hindered by the currently undefined diagnostic criteria. However, with the proposal to include the requirement of standing SBP reaching ≥140 mmHg (Jordan et al., 2023), true prevalence could be lower than what the literature currently alludes to. Based on a 20 mmHg increase from baseline, a recent study on orthostatic hypertension and frailty found marginally higher rates of orthostatic hypertension compared with their orthostatic hypotension group (Choi et al., 2024). Likewise, our study indicated higher orthostatic hypertension rates, as a measure of the OHYPER cluster size, than their OHYPO cluster counterpart. This observation could be significant, because, like orthostatic hypotension, preliminary studies on orthostatic hypertension also indicate a higher incidence of cardiovascular disease and all‐cause mortality in this cohort (Juraschek et al., 2023; Kostis et al., 2019).

### 4.4 Artifical intelligence and beat‐to‐beat cardiovascular data

Although it is generally argued that the magnitude and duration of blood pressure drops are most relevant to worse outcomes, including mortality risk (Frith et al., 2016; Wiersinga et al., 2022), delayed blood pressure overshoots in orthostatic hypertension could be more meaningful. The OHYPER cluster showed marginal early blood pressure increases, with more pronounced rises beyond 40 s of the orthostatic challenge. This haemodynamic profile of our OHYPER cluster members and their poorer frailty outcomes are consistent with observations by Roca et al. (2022), who observed slower gait speed with later‐phase blood pressure increases at 1 and 2 min after standing. A community‐based study demonstrated that SBP elevations occurring at 3 min and not in the first minute of standing were associated with higher all‐cause mortality (Velilla‐Zancada et al., 2017). This contrasts with a relationship between blood pressure recovery deficits in orthostatic hypotension within the first minute and excess mortality in falls clinic patients (Lagro et al., 2014). The broad interpretation of differential features suggests that with the appropriate methods (currently, AI appears most capable) there is great prognostic value in risk profiling dysregulated postural blood pressure into distinct haemodynamic profiles.

Continuous non‐invasive cardiovascular data, rich in detail, capture the intricacies of the haemodynamic response to postural change. *k*‐means clustering, an unsupervised machine learning method, is aptly suited to interpret these data. Moreover, this technique accounts for both increases and decreases in blood pressure from the baseline, an essential consideration in measuring the rises observed in emergent orthostatic hypertension. An earlier effort to apply *k*‐means clustering to beat‐to‐beat data for analysing postural blood pressure was made by Romero‐Ortuno, Cogan, Foran, et al. (2011), followed by a single replication by researchers who adopted the same approach (Cooke et al., 2013). However, these studies had relatively small sample sizes and did not specify feature variables informed by the morphologies of recognized conditions of dysregulated postural blood pressure. Furthermore, the *k*‐means++ algorithm, an advancement from *k*‐means designed to create more distinct clusters, was not used. These factors could have contributed to the morphologies of their clusters not closely mirroring defined disorders of postural blood pressure regulation.

More than a decade after these initial attempts, our study resumes at a point when advanced AI models are poised rapidly to accelerate our interpretation of complex data. For instance, a deep learning model applied to continuous ECG monitoring showed enhanced accuracy in predicting atrial fibrillation with extended atrial fibrillation‐free data windows (Gadaleta et al., 2023). This highlights the notion that although conventional analysis might identify the expected features of a conditions, AI can detect these attributes in a signal where they are not readily apparent.

### 4.5 Limitations

Our work has its limitations. This research is a retrospective cross‐sectional analysis, limiting the exploration of a temporal relationship between dysregulated postural blood pressure and outcomes. Our data were obtained during the active stand test, which can be prone to noise and variability. The reliability of orthostatic beat‐to‐beat blood pressure assessments has come under scrutiny (Finucane, Savva, & Kenny, 2017), primarily driven by inquiry into the calibration of finger‐ to brachial‐level blood pressures and the relevance of subtle blood pressure changes. Additionally, there is known within‐person variability, which ranges from diurnal rhythm (Ward & Kenny, 1996) to seasonal shifts (Weiss et al., 2006). Besides inherent natural variability, reproducibility is challenged from the very start of the data collection phase during the active stand test, where replicating the same transition each time is unachievable. The MELoR study protocol adopted an approach to consistency in the active stand throughout the data collection phase, and the beat‐to‐beat data were screened further for quality in the custom‐designed signal quality framework. The presented results were not compared with consensus‐derived diagnostic thresholds for postural blood pressure conditions, nor were intermittent brachial measurements cross‐referenced against the cluster morphologies. It is argued that intermittent sphygmomanometer measurements might under‐report postural blood pressure conditions, whereas beat‐to‐beat methods might over‐report them. However, a study indicated that despite moderately low concordance between these methods, the prevalence of orthostatic hypotension diagnosis was not significantly different (Breeuwsma et al., 2018).

There remain some unanswered questions in our results, especially regarding the favourable outcomes in clusters with blood pressure drops. Orthostatic hypotension is frequently encountered in general medical practice and can present with symptoms of blurred vision, weakness, dizziness, fatigue, coat‐hanger headache and ‘brain fog’ (Kaufmann et al., 2012). These symptoms could be anticipated to lead to progressive functional decline and deconditioning, accompanied by a diminished quality of life. Given this general clinical perspective on orthostatic hypotension, the modest yet superior activities of daily living scores and improved Fried's frailty in the OHYPO cluster within our cohort are intriguing and merit further investigation.

We defined our methodology and selection of three variables for the feature vectors in the beat‐to‐beat data because they met the challenges of AI explainability and domain knowledge and allowed for an intuitive three‐dimensional visualization of the clustered data points. The decision to limit our clustering to these features, chosen a priori, could overlook time points with greater clinical relevance. Furthermore, impedance cardiography was not used concurrently to monitor cardiac function during blood pressure assessments. This technology could have enriched our analysis with key metrics, such as cardiac output, stroke volume and total peripheral resistance. These indices would offer additional insight into the pathophysiological underpinnings of the postural blood pressure profiles and frailty outcomes observed in our research.

### 4.6 Future research and wider impact

The potential for future research and the broader impact of our work resides in unlocking the information held within postural blood pressure profiles. These profiles could offer two key applications, evident through their associations with the frailty outcomes of our study. First, they could be instrumental in predicting the risk of outcomes and adverse events specific to their morphologies, and second, they could act as markers of overall physiological health. Postural change activates the cardiovascular and autonomic nervous systems, providing valuable insight into their functioning. Linking the blood pressure profile to its underlying pathophysiology in its respective system would enable more personalized treatment approaches (Owen et al., 2022), with broader prognostic health and disease‐monitoring applications at the population level.

Recently, the American Autonomic Society and the Japanese Society of Hypertension have endorsed a proposal defining orthostatic hypertension as a 20 mmHg increase from baseline blood pressure coupled with a standing blood pressure of ≥140 mmHg. Additionally, the experts recommend a careful reframing of the terminology, introducing an ‘exaggerated orthostatic pressor response’ to describe individuals who do not meet all criteria for orthostatic hypertension (Jordan et al., 2023). As evidence is released to support orthostatic hypertension as a distinct entity, the evolving terminology poses a challenge to consistent between‐study comparison. We recognize that the criteria and terminology will be likely to evolve based on insights from forthcoming research. In light of such developments, future work could expand beyond blood pressure recorded during orthostatic challenge, exploring longer‐term pressures in those with orthostatic hypertension.

Investigators could continue to explore the causal pathways of dysregulated postural blood pressure using more sophisticated AI methods. It is unknown whether currently defined blood pressure morphologies signal underlying pathophysiology or are outcomes of progressive end‐organ damage as a consequence of the conditions themselves. Interventional and population‐based studies rarely report on orthostatic hypertension, despite increasing acknowledgement of its negative association with a range of outcomes.

The decision on the number of clusters (*k*) was supported by metrics from cluster quality assessments, together with the aim to accommodate both orthostatic hypotension and orthostatic hypertension morphologies. Although a two‐cluster model showed marginal improvement in stability compared with the three‐cluster model, to this end we prioritized exploring the larger cluster representation. Future research could investigate the optimal number of clusters that can be represented effectively in such models, supported by clinical associations. This approach would fulfil the objectives of identifying postural blood pressure patterns that are both easily interpretable in clinical settings and derived from validated clustering models.

Although our study focused on frailty, further research evaluating orthostatic hypertension in the context of cardiovascular and autonomic function, using indicators available in beat‐to‐beat cardiovascular data, could enrich the existing evidence base and deepen our understanding of orthostatic hypertension.

## 5 CONCLUSION

Using a machine learning model, our study identified postural blood pressure profiles from beat‐to‐beat data associated with frailty outcomes. Profiles characterized by drops in blood pressure upon standing were independently linked to more favourable outcomes, whereas orthostatic hypertension had the poorest frailty performance. Orthostatic hypertension, still in its infancy of being defined in diagnostic and treatment guides, is gaining attention as clinically meaningful owing to the growing evidence base of associated adverse outcomes. The variation in outcomes across our clusters suggests that there are distinct frailty phenotypes for orthostatic hypotension and orthostatic hypertension.

## AUTHOR CONTRIBUTIONS

Initial concept design, application for data acquisition, analysis and original draft by Claire M. Owen. First draft validation by James Frith and Jaume Bacardit. Interpretation of the results, revisions and edits were made collaboratively by all authors (Claire M. Owen, Jaume Bacardit, Maw P. Tan, Nor I. Saedon, Choon‐Hian Goh, Julia L. Newton and James Frith). All authors have approved the final manuscript version and commit to addressing any questions to uphold the accuracy and integrity of the work. All individuals listed as authors meet the authorship criteria, and all who qualify for authorship are listed.

## CONFLICT OF INTEREST

The authors declare no conflicts of interest.

## FUNDING INFORMATION

None.

## Data Availability

The data underlying this article cannot be shared publicly owing to the privacy of individuals who participated in the study.
